# Intraoperative arteriovenous patient sampling to assess in situ non–small cell lung cancer metabolism

**DOI:** 10.1172/JCI198821

**Published:** 2026-01-27

**Authors:** Johnathan R. Kent, Keene L. Abbott, Rachel Nordgren, Amy Deik, Nupur K. Das, Millenia Waite, Tenzin Kunchok, Anna Shevzov-Zebrun, Nathaniel Christiansen, Amir Sadek, Darren S. Bryan, Mark K. Ferguson, Jessica S. Donington, Alexander Muir, Yatrik M. Shah, Clary B. Clish, Matthew G. Vander Heiden, Maria Lucia L. Madariaga, Peggy P. Hsu

**Affiliations:** 1 Department of Surgery, University of Chicago, Chicago, Illinois, USA.; 2 Department of Biology and Koch Institute for Cancer Research, Massachusetts Institute of Technology, Cambridge, Massachusetts, USA.; 3 Broad Institute, Cambridge, Massachusetts, USA.; 4 Department of Public Health Sciences, University of Chicago, Chicago, Illinois, USA.; 5 Department of Molecular and Integrative Physiology, University of Michigan, Ann Arbor, Michigan, USA.; 6 Whitehead Institute for Biomedical Research, Cambridge, Massachusetts, USA.; 7 Ben May Department of Cancer Research, University of Chicago, Chicago, Illinois, USA.; 8 Department of Internal Medicine, and; 9 Rogel Cancer Center, University of Michigan, Ann Arbor, Michigan, USA.; 10 Dana-Farber Cancer Institute, Boston, Massachusetts, USA.

**Keywords:** Metabolism, Oncology, Glucose metabolism, Lung cancer, Metabolomics

## Abstract

Here authors report intraoperative sampling of blood entering and leaving the lung allowing direct assessment of nutrient use by lung cancer.

**To the Editor:** Alterations in metabolism are a hallmark of cancer (1). While stable isotope tracing and tissue metabolite measurements have provided insights into tumor metabolism, these approaches do not directly measure metabolite consumption and secretion. Furthermore, human studies often rely on cell lines, whose metabolism is influenced by media conditions, or on surgical specimens, in which metabolic integrity is compromised by prolonged arterial ligation. We sought to gain deeper insights into lung cancer metabolism by intraoperative sampling, which avoids these pitfalls.

We evaluated non–small cell lung cancer (NSCLC) metabolism by sampling arterial inflow and venous outflow of tumor-bearing versus non-tumor-bearing lung tissue in patients during 20 lung cancer resections (Supplemental Figure 1, A–E, and Supplemental Table 1; supplemental material available online with this article; https://doi.org/10.1172/JCI198821DS1). Most tumors were adenocarcinomas (90%), half were stage I, and 25% had been treated with neoadjuvant therapy (Supplemental Figure 1F). Blood was collected intraoperatively from the pulmonary artery, pulmonary veins draining tumor-bearing and non-tumor-bearing lung lobes, and from a radial arterial line and a peripheral vein to characterize systemic circulation (Supplemental Figure 1, A–E). To confirm metabolite stability under intraoperative handling conditions, mock samples were processed and quantified by liquid chromatography–mass spectrometry (LC-MS) using a library of chemical standards (2); >97% of polar metabolites remained stable (Supplemental Figure 1, G–I). No intraoperative bleeding or complications resulted from pulmonary vasculature sampling.

We next analyzed all intraoperative samples using the same LC-MS approach. To assess normal lung metabolism, we compared concentration changes in the systemic circulation (peripheral vein versus radial artery) with those across non-tumor-bearing lungs (pulmonary vein versus pulmonary artery). Principal component analysis (PCA) showed a clear separation between systemic and lung circulation (Figure 1A), with many metabolites exhibiting differential utilization (Figure 1B and Supplemental Figure 2A). Healthy lung tissue secreted lower levels of lactate, pyruvate, α-ketoglutarate (AKG), citrate, and fumarate and consumed less glucose, glutamate, and citrulline compared with the systemic circulation (Figure 1, C–E), consistent with the lung’s relatively low metabolic activity (3).

We next examined tumor-bearing lungs. Non-normalized venous concentrations showed no significant differences between tumor-bearing and normal lungs (Supplemental Figure 2, B and C). However, after normalizing venous values to each participant’s pulmonary artery concentrations — an approach that reduces interindividual variability — clear metabolic differences emerged (Figure 1, F–H, and Supplemental Figure 2D). These patterns persisted when we restricted analysis to participants without neoadjuvant therapy (*n* = 15) (Supplemental Figure 3, A–C), underscoring the sensitivity of arteriovenous sampling relative to peripheral blood analyses.

Lactate emerged as a top metabolite secreted at higher levels in tumor-bearing lungs, whereas glucose consumption was similar between groups (Figure 1I). Succinate, fumarate, and glycine levels were also elevated or trended higher in the tumor-draining veins. These effects were somewhat attenuated when excluding patients who received neoadjuvant therapy, but evidence for increased lactate secretion remained significant. (Supplemental Figure 3D). No consistent differences were observed for lipid or metal species (Supplemental Figure 3, E and F). Notably, absolute lactate secretion correlated with tumor PET avidity but not size (Figure 1J and Supplemental Figure 3G); however, because these are absolute concentrations, they may be influenced by systemic levels, blood flow, and/or clearance and thus do not directly reflect glycolytic flux.

These findings help refine existing paradigms of lung cancer metabolism. Prior studies have shown that a subset of NSCLC tumors can incorporate lactate carbon into the TCA cycle (4). In our cohort, tumor-bearing lung tissue displayed marked heterogeneity, with only a subset of tumors showing clear net lactate export, consistent with enhanced glycolysis in some tumors. This variability parallels isotope-labeling studies, in which only a subset of NSCLCs exhibited lactate utilization, and most were inferred to have net lactate export. Apparent differences likely reflect methodological differences, as isotope tracing reports carbon incorporation but cannot determine net metabolite consumption or secretion. Although previous studies have suggested aspartate as a limiting metabolite for cancer cells (5), we observed higher aspartate levels in the tumor-draining pulmonary vein, suggesting that aspartate availability and utilization by tumors may be context dependent.

Our work underscores the value of direct in vivo approaches to characterize cancer metabolism and demonstrates the utility of intraoperative arteriovenous sampling for quantifying nutrient fluxes across tumor-bearing organs. Limitations include the small cohort size and the need to compare flux measurements with tissue-based analyses. Additionally, we cannot exclude the possibility that cancer alters the metabolism of adjacent lung tissue within the tumor-bearing lobe.

## Funding support

This work is the result of NIH funding, in whole or in part, and is subject to the NIH Public Access Policy. Through acceptance of this federal funding, the NIH has been given a right to make the work publicly available in PubMed Central.

- National Science Foundation (DGE-1122374, to KLA).
- NIH (F31CA271787 and T32GM007287, to KLA; R01CA148828 and R01CA245546, to YMS; R35CA242379 and P30CA1405141, to MGVH; and K08CA286759, to PPH).
- MGVH acknowledges support from the MIT Center for Precision Cancer Medicine (to MGVH).
- Ludwig Center at MIT (to MGVH).
- Respiratory Health Association (to MLLM).
- Rogel Cancer Center at the University of Michigan (to PPH).
- Judith Tam ALK Research Initiative (to PPH).
- Lung Cancer Research Foundation (to PPH).
- Burroughs Wellcome Fund Career Award for Medical Scientists (to PPH).

## Supplementary Material

Supplemental data

Supporting data values

## Figures and Tables

**Figure 1:**
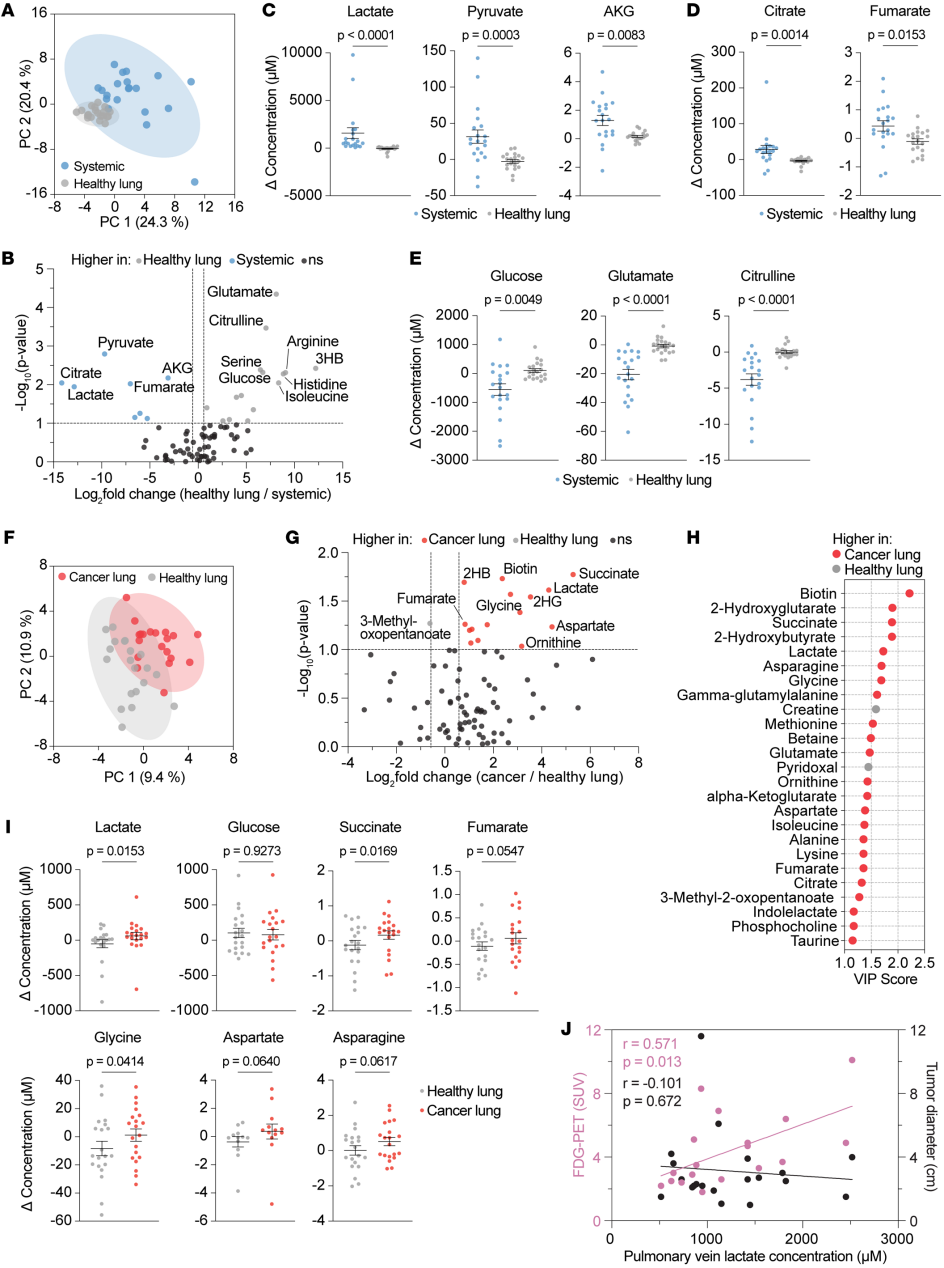
Intraoperative arteriovenous sampling reveals nutrient consumption and production in healthy versus tumor-bearing lung tissue. (**A**) Partial least-squares discriminant analysis (PLS-DA) comparing metabolite profiles for healthy lung circulation (pulmonary vein of a nontumor lobe minus the pulmonary artery) versus systemic circulation (peripheral vein minus the radial artery). (**B**) Volcano plot of metabolites differing between healthy lung and systemic circulation (fold change >1.5; *P* < 0.1, by paired, 2-tailed *t* test; *n* = 20). 3HB, 3-hydroxybutyrate. (**C**–**E**) Selected metabolites that are elevated (**C** and **D**) or reduced (**E**) in systemic versus healthy lung circulation. Data indicate the mean ± SEM. *P* values were determined by Wilcoxon matched-pairs, signed-rank test (*n* = 20). (**F**) PLS-DA of cancer-bearing versus healthy lung circulation (pulmonary vein minus pulmonary artery). (**G**) Volcano plot of metabolites differing between cancer-bearing and healthy lungs (fold change >1.5; *P* < 0.1, by paired, 2-tailed *t* test; *n* = 20). 2HB, 2-hydroxybutyrate; 2HG, 2-hydroxyglutarate. (**H**) Variable importance in projection (VIP) plot of the top 25 metabolites distinguishing cancer and healthy lungs, derived from **F**. (**I**) Selected metabolites differing between cancer-bearing and healthy lungs. Data indicate the mean ± SEM. *P* values were determined by Wilcoxon matched-pairs, signed-rank test (*n* = 20). (**J**) Lactate concentration in the cancer-draining pulmonary vein plotted against fluorodeoxyglucose-PET (FDG-PET) standardized uptake value (SUV) (magenta, *n* = 18) or tumor diameter (black, *n* = 20). Pearson’s *r* and *P* values are shown.

## Version 1 01/27/2026

In-Press Preview

## Version 2 03/16/2026

Electronic publication
