# Supplementary material for: Intraoperative arteriovenous patient sampling to assess in situ non–small cell lung cancer metabolism
Source: J Clin Invest. 2026 Jan 27;136(6):e198821. doi: 10.1172/JCI198821 (PMC12987651; doi:10.1172/JCI198821)
Supplement: Supplemental data [file jci-136-198821-s334.pdf]

Supplementary Material

Participant	Age	Sex	Race	Tumor Location	Histology	Neoadjuvant Therapy	Pathology Tumor Size (cm)	Pathologic Stage	Comorbidities
1	66	F	Caucasian	RML	Adeno	None	2.8	pT1cN0	Breast Cancer, HLD, HTN
2	76	F	Caucasian	RLL	Adeno	Carboplatin + Pemetrexed	2.8	ypT2aN2	HLD, HTN
3	81	F	Caucasian	RUL	Adeno	None	3.1	pT1cN0	Breast Cancer, Hypothyroidism, Raynaud's disease
4	62	M	Caucasian	RUL	Adeno	None	3.5	pT2aN0	BPH
5	67	M	African American	LLL	Adeno	None	1.9	pT1bN1	Aortic stenosis, CKD, COPD, DM, Gout, HLD, HTN
6	70	F	Caucasian	RML	Adeno	None	1.8	pT1bN1	CAD, Hypothyroid, HLD, HTN, MDD, OA
7	74	F	Caucasian	LUL	Adeno	Clinical Trial: Cisplatin + Pemetrexed +/- Nivolumab	0	ypT0N0M0	DM, HLD, HTN, IBS, TIA
8	79	F	Caucasian	RML	Adeno	None	0.9	pT1aN0	Breast Cancer, Lymphoma, HLD, HTN, hypothyroid
9	73	F	Caucasian	RLL	Adeno	Cisplatin + Pemetrexed	2.2	ypT1cN0	HLD, HTN, OA
10	67	M	Caucasian	LUL	SCC	Clinical Trial: Cisplatin + Pemetrexed +/- Nivolumab	0.1	ypT1aN0	HLD
11	69	M	Caucasian	RUL	Adeno	None	5.7	pT3N0	DM, HTN
12	28	F	African American	LUL	Adeno	None	1.5	pT2aN1	IBS, Iron deficiency anemia, Li-Fraumeni syndrome
13	76	F	Caucasian	LUL	Adeno	None	2.6	pT1cN0	Age Related Macular Degeneration , DM, HTN, HLD,
14	77	F	African American	LLL	Adeno	None	1	pT3N0	Diverticulosis, HLD, HT
15	73	F	African American	RLL	SCC	None	3.5	pT2aN2	Anxiety, GERD, HTN, MDD, OA
16	77	M	Caucasian	LLL	Adeno	None	1.5	pT1bN0	HLD, Rectal Adenocarcinoma
17	67	F	Caucasian	LUL	Adeno	Carboplatin + Paclitaxel + Pembrolizumab	2.9	ypT3N0	CAD, GERD, HLD, Thrombocytopenia

18*	47	F	Other	LUL	Adeno	None	3.2	pT1bN0	DM, HLD, HTN, OA, OSA
19*	47	F	Other	RLL	Adeno	None	3.8	pT2aN0	DM, HLD, HTN, OA, OSA
20	49	F	Caucasian	RUL	Adeno	None	2.3	pT1cN0	Asthma, GERD, HTN

Abbreviations: M – Male; F- Female; RUL – Right Upper Lobe; RML – Right Middle Lobe; RLL – Right Lower Lobe; LUL – Left Upper Lobe; LLL – Left Lower Lobe; Adeno – Adenocarcinoma; SCC – Squamous Cell Carcinoma; BPH – Benign Prostatic Hypertrophy; CAD – Coronary Artery Disease; COPD – Chrnoic Obstructive Pulmonary Disease; CKD – Chronic Kidney Disease; DM – Diabetes Mellitus; GERD – Gastroesophageal Refulx Disease; HLD – Hyperlipidemia; HTN – Hypertension; IBS - Irritable Bowel Syndrome; MDD – Major Depressive Disorder; OA -Osteoarthritis; OSA – Obstructive Sleep Apnea; TIA – Transient Ischemic Attack.
* Subject 18/19 was the same patient with cosynchronous bilateral tumors that were resected in separate encounters

Supplemental Table 1. Clinical and pathological characteristics of study participants. Summary of demographic, clinical and pathological data of 20 included non-small cell lung cancer resections from 19 patients.

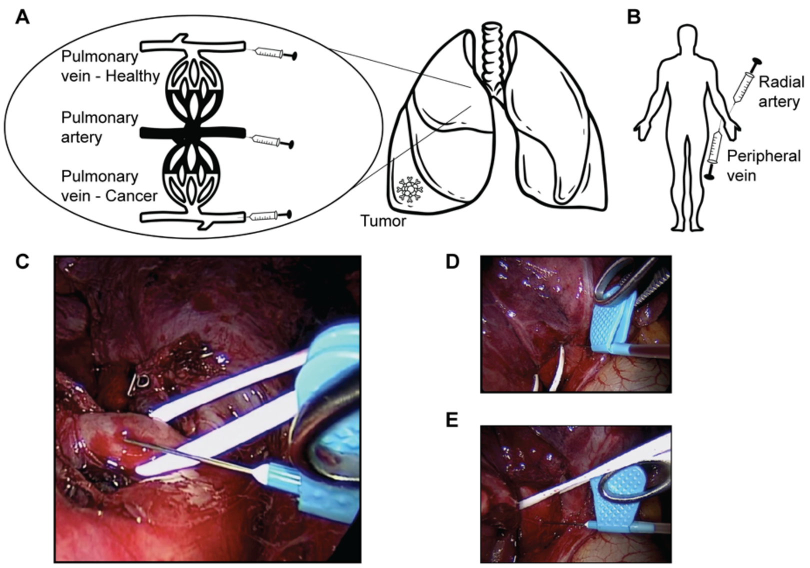

F

Participants	n = 20	
Age (yr)	66.8 ± 13.7	
Sex	Female	75% (n = 15)
Smoking status	Former	65% (n = 13)
	Never	25% (n = 5)
	Current	10% (n = 2)
Neoadjuvant chemotherapy	Yes	25% (n = 5)
Histology	Adenocarcinoma	90% (n = 18)
	Squamous cell carcinoma	10% (n = 2)
Tumor size (cm)	3.1 ± 2.3	
SUV	4.4 ± 2.2	

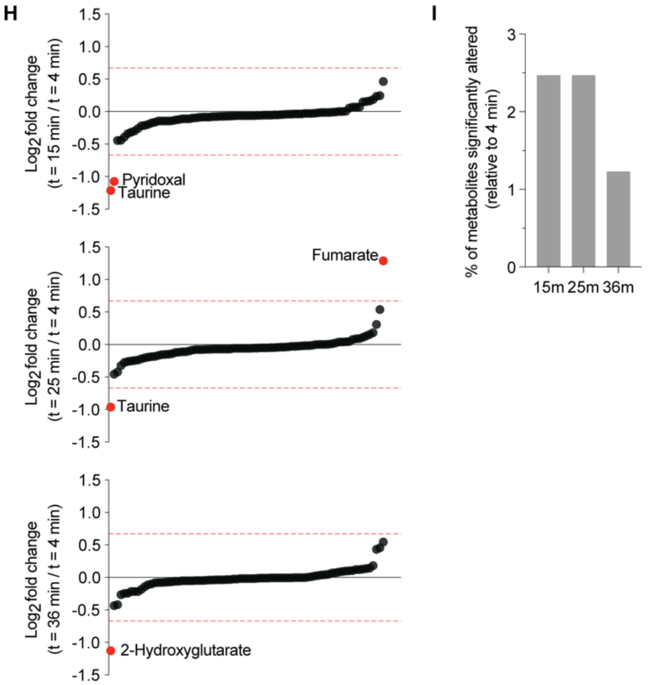

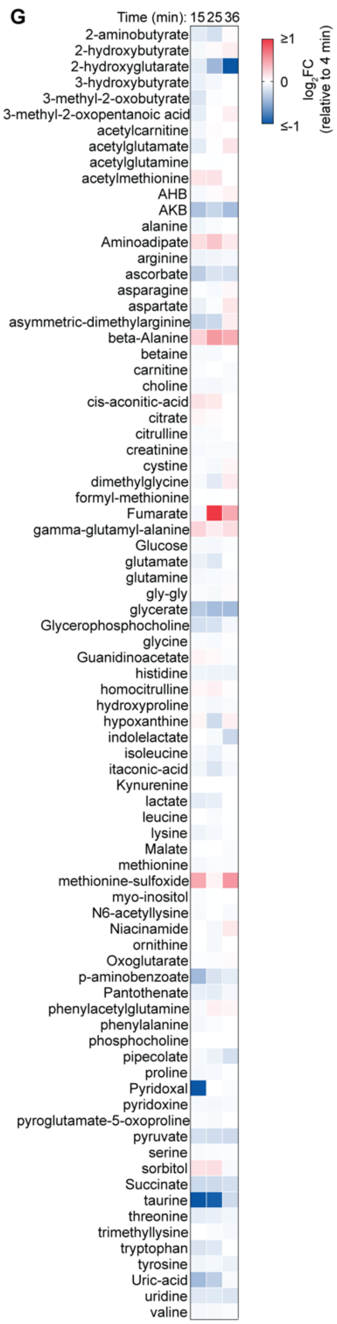

Supplemental Figure 1. Validation of intraoperative blood sampling and processing conditions. (A-B) Schematic illustrating sampling sites in the study. (A), Pulmonary vasculature including tumor-bearing and non-tumor-bearing (healthy) lung lobes. (B), Systemic sites including the radial artery and a peripheral vein. **(C-E)** Intraoperative imaging during a video assisted thoracoscopic right upper lobectomy. Sampling of the pulmonary artery (C), pulmonary vein draining the cancer containing lobe (right superior pulmonary vein) (D); and healthy lobe (right middle pulmonary vein) (E) is shown. **(F)** Summary of clinical characteristics for the 20 NSCLC participants included in the study. **(G)** Heatmap showing average $\log_2$ fold changes in plasma metabolite concentrations over the sample processing time, measured relative to baseline (t = 4 min). AHB: alpha-hydroxybutyrate; AKB: alpha-ketobutyrate; gly-gly: glycyl-glycine. **(H)** Plot showing ranked metabolites based on absolute $\log_2$ fold change at different times during sample processing as indicated. Metabolites exceeding the significance threshold ($|\log_2$ fold change$| > 0.58$) at each time point are highlighted in red. **(I)** Bar plot showing the percentage of metabolites significantly altered ($|\log_2$ fold change$| > 0.58$) at each time point during sample processing relative to t = 4 min.

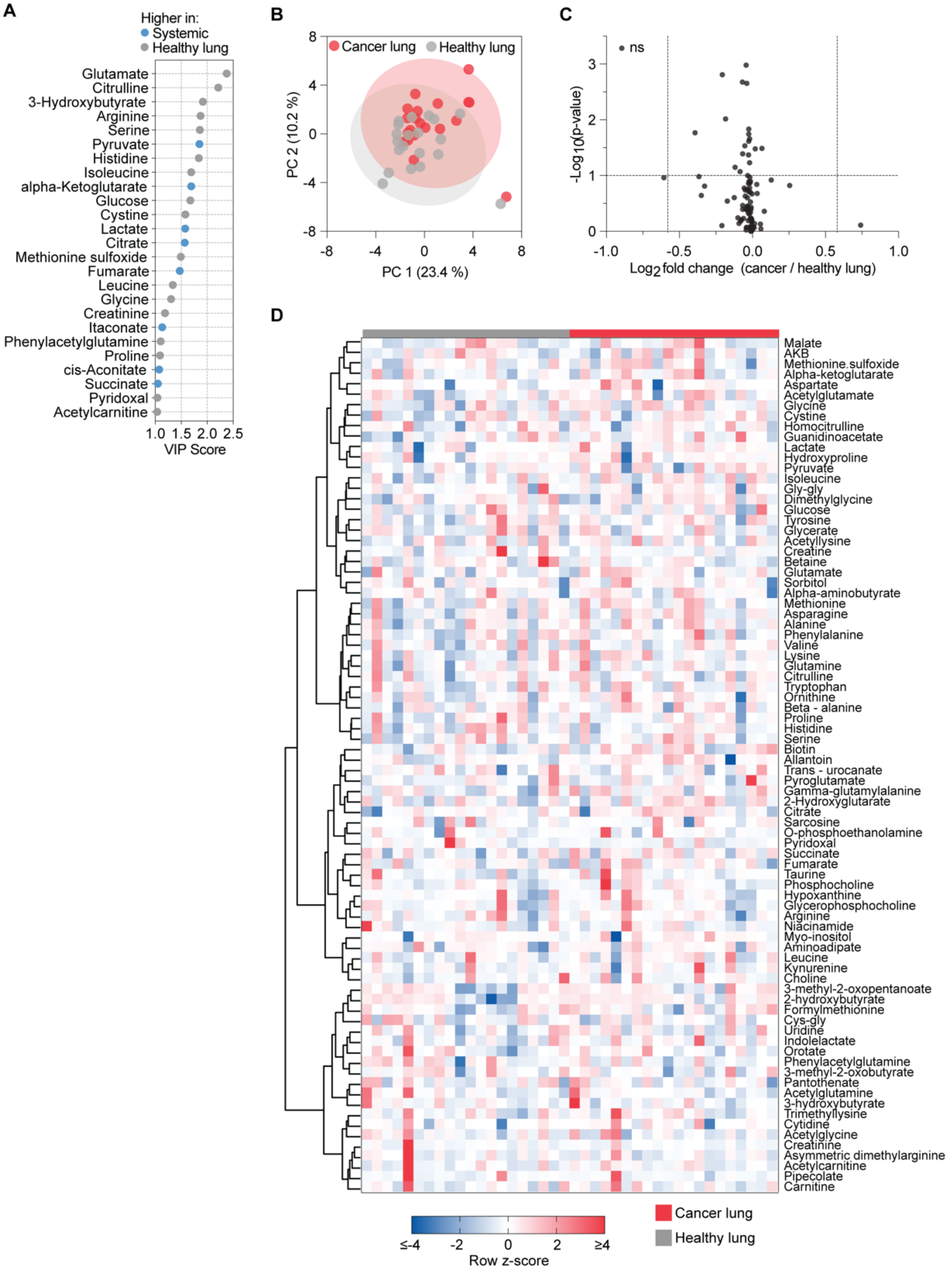
A
Higher in:
Systemic
Healthy lung
Glutamate
Citrulline
3-Hydroxybutyrate
Arginine
Serine
Pyruvate
Histidine
Isoleucine
alpha-Ketoglutarate
Glucose
Cystine
Lactate
Citrate
Methionine sulfoxide
Fumarate
Leucine
Glycine
Creatinine
Itaconate
Phenylacetylglutamine
Proline
cis-Aconitate
Succinate
Pyridoxal
Acetylcarnitine
1.0 1.5 2.0 2.5
VIP Score
B
Cancer lung
Healthy lung
PC 2 (10.2 %)
PC 1 (23.4 %)
8 4 0 -4 -8
-8 -4 0 4 8
C
ns
-Log10(p-value)
Log2 fold change (cancer / healthy lung)
3 2 1 0
-1.0 -0.5 0.0 0.5 1.0
D
Malate
AKB
Methionine.sulfoxide
Alpha-ketoglutarate
Aspartate
Acetylglutamate
Glycine
Cystine
Homocitrulline
Guanidinoacetate
Lactate
Hydroxyproline
Pyruvate
Isoleucine
Gly-gly
Dimethylglycine
Glucose
Tyrosine
Glycerate
Acetyllysine
Creatine
Betaine
Glutamate
Sorbitol
Alpha-aminobutyrate
Methionine
Asparagine
Alanine
Phenylalanine
Valine
Lysine
Glutamine
Citrulline
Tryptophan
Ornithine
Beta - alanine
Proline
Histidine
Serine
Biotin
Allantoin
Trans - urocanate
Pyroglutamate
Gamma-glutamylalanine
2-Hydroxyglutarate
Citrate
Sarcosine
O-phosphoethanolamine
Pyridoxal
Succinate
Fumarate
Taurine
Phosphocholine
Hypoxanthine
Glycerophosphocholine
Arginine
Niacinamide
Myo-inositol
Aminoadipate
Leucine
Kynurenine
Choline
3-methyl-2-oxopentanoate
2-hydroxybutyrate
Formylmethionine
Cys-gly
Uridine
Indolelactate
Orotate
Phenylacetylglutamine
3-methyl-2-oxobutyrate
Pantothenate
Acetylglutamine
3-hydroxybutyrate
Trimethyllysine
Cytidine
Acetylglycine
Creatinine
Asymmetric dimethylarginine
Acetylcarnitine
Pipecolate
Carnitine
≤-4 -2 0 2 ≥4
Row z-score
Cancer lung
Healthy lung

Supplemental Figure 2. Direct comparison of pulmonary vein metabolite levels lacks sensitivity to distinguish cancer from healthy lung. (A) Variable importance in projection (VIP) plot of the top 25 metabolites distinguishing non-tumor-bearing (healthy) lung circulation (pulmonary vein of a non-tumor lobe minus pulmonary artery) from systemic circulation (peripheral vein minus radial artery). Metabolites in blue are elevated in systemic circulation; those in grey are elevated in healthy lung. Derived from data shown in Figure 1B. **(B)** Partial least squares discriminant analysis (PLS-DA) comparing metabolite profiles from the pulmonary vein of a healthy lobe versus that of a cancer-bearing lobe (n = 20). **(C)** Volcano plot depicting metabolites significantly altered between cancer-bearing and healthy pulmonary veins. Significance was defined as a fold change >1.5 and a raw p-value <0.1 (paired two-tailed t-test, n = 20). **(D)** Hierarchical clustering of z-score–normalized metabolite concentrations in blood from cancer-bearing versus healthy lung lobes (both calculated as pulmonary vein minus pulmonary artery). Rows represent individual metabolites; patient groups were not clustered (n = 20). AKB: alpha-ketobutyrate; cys-gly: cysteinylglycine; gly-gly: glycyl-glycine.

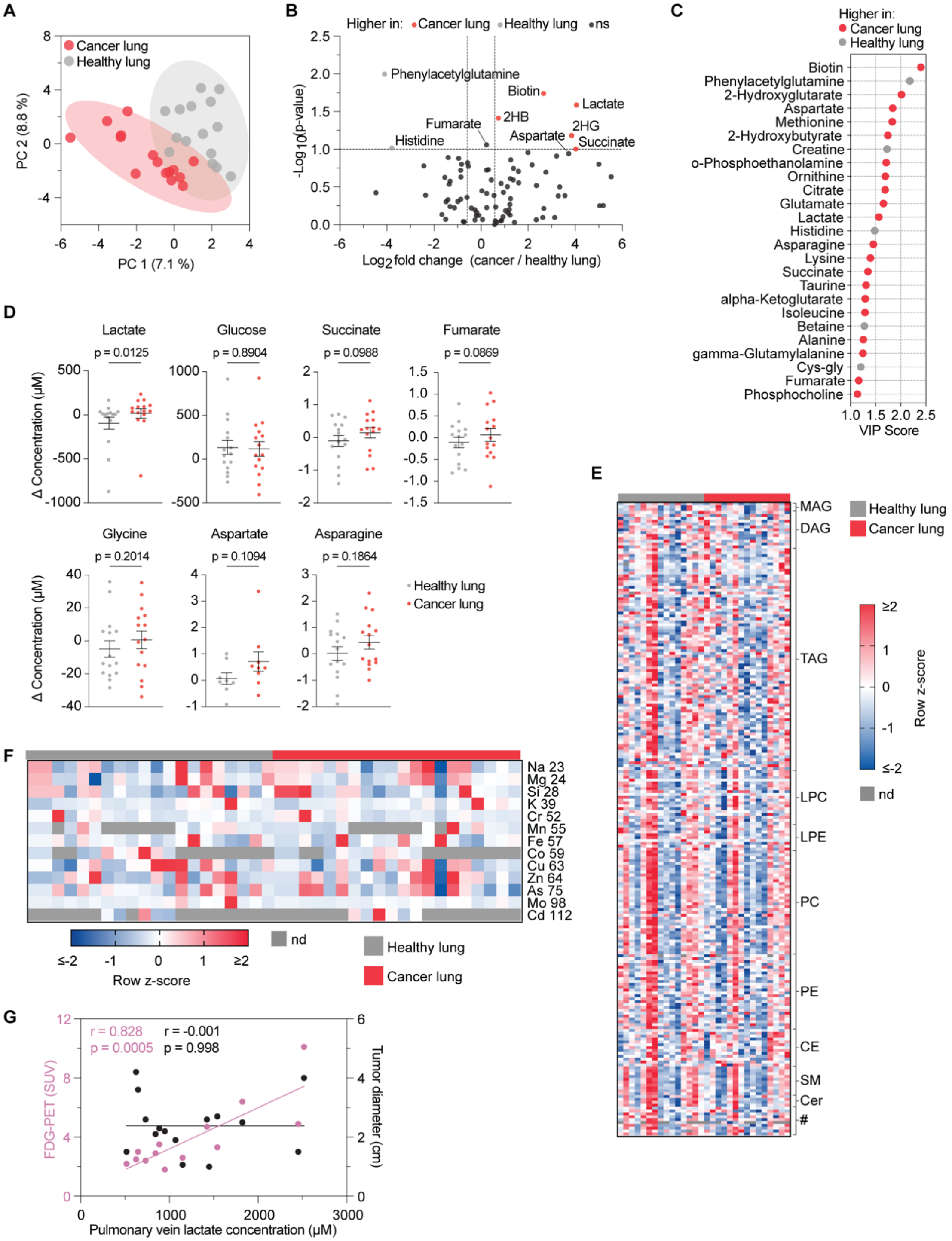
A
Cancer lung
Healthy lung
PC 2 (8.8 %)
PC 1 (7.1 %)
B
Higher in: Cancer lung Healthy lung ns
Phenylacetylglutamine
Biotin
Lactate
2HB
Fumarate
2HG
Histidine
Aspartate
Succinate
-Log10(p-value)
Log2 fold change (cancer / healthy lung)
C
Higher in:
Cancer lung
Healthy lung
Biotin
Phenylacetylglutamine
2-Hydroxyglutarate
Aspartate
Methionine
2-Hydroxybutyrate
Creatine
o-Phosphoethanolamine
Ornithine
Citrate
Glutamate
Lactate
Histidine
Asparagine
Lysine
Succinate
Taurine
alpha-Ketoglutarate
Isoleucine
Betaine
Alanine
gamma-Glutamylalanine
Cys-gly
Fumarate
Phosphocholine
VIP Score
D
Lactate
p = 0.0125
Glucose
p = 0.8904
Succinate
p = 0.0988
Fumarate
p = 0.0869
Δ Concentration (μM)
Glycine
p = 0.2014
Aspartate
p = 0.1094
Asparagine
p = 0.1864
Healthy lung
Cancer lung
E
MAG
DAG
TAG
LPC
LPE
PC
PE
CE
SM
Cer
#
Healthy lung
Cancer lung
≥2
1
0
-1
≤-2
Row z-score
nd
F
Na 23
Mg 24
Si 28
K 39
Cr 52
Mn 55
Fe 57
Co 59
Cu 63
Zn 64
As 75
Mo 98
Cd 112
≤-2 -1 0 1 ≥2
Row z-score
nd
Healthy lung
Cancer lung
G
r = 0.828
p = 0.0005
r = -0.001
p = 0.998
FDG-PET (SUV)
Tumor diameter (cm)
Pulmonary vein lactate concentration (μM)

Supplemental Figure 3. Tumor-associated metabolic alterations are also found in patients who did not receive neoadjuvant therapy. (A) Partial least squares discriminant analysis (PLS-DA) comparing metabolite profiles from cancer-bearing versus non-cancer bearing (healthy) lung tissue (both calculated as pulmonary vein minus pulmonary artery) in patients who did not receive neoadjuvant therapy (n = 15). **(B)** Volcano plot showing metabolites significantly altered between cancer-bearing and healthy lung in this subset of patients. Significance defined as fold change >1.5 and raw p < 0.1 (paired two-tailed t-test, n = 15). 2HB: 2-Hydroxybutyrate; 2HG: 2-Hydroxyglutarate. **(C)** Variable importance in projection (VIP) plot of the top 25 metabolites distinguishing cancer from healthy lung in patients without neoadjuvant therapy. Metabolites in red are elevated in cancer; those in grey are elevated in healthy lung. Based on data in panel (A). **(D)** Selected metabolites differing between cancer-bearing and healthy lung in patients who did not receive neoadjuvant therapy. Data are mean ± SEM; p-values from Wilcoxon matched-pairs signed rank test (n = 15). **(E)** Heatmap of lipid species in the peripheral vein draining healthy (grey) or cancer-bearing (red) lung, normalized to the pulmonary artery. Each row is z-score normalized across samples (n = 15). Lipid classes: MAG, monoacylglyerol; DAG, diacylglycerol; TAG, triacylglycerol; LPC, lysophosphatidylcholine; LPE, lysophosphatidylethanolamine; PC, phosphatidylcholine; PE, phosphatidylethanolamine; CE, cholesteryl ester; SM, sphingomyelin; Cer, ceramide. @ denotes the following metabolites (top to bottom): sphingosine, palmitoylethanolamide, cholesterol, campesterol, piperine, coenzyme Q9, coenzyme Q10, alpha-tocopherol, delta-tocopherol, gamma-tocopherol. **(F)** Heatmap of metal ion concentrations in the pulmonary vein draining healthy (grey) and cancer-bearing (red) lung lobes, normalized to the pulmonary artery. Data represent all participants (n = 20). Each row is z-score normalized across samples. **(G)** Scatter plot showing lactate levels in the cancer-draining pulmonary vein plotted against FDG-PET SUV (magenta, n = 13) or tumor size size (black, n = 15) in patients that did not receive neoadjuvant therapy. Pearson correlation coefficients and p-values are indicated.

Methods

Patient selection

Patients with resectable biopsy-proven non-small cell lung cancer with tumors greater than 1cm on pre-operative imaging were approached for participation in the general thoracic surgery clinic at University of Chicago Medical Center from July 2021 – May 2023. Patients were excluded if they had a history of inherited metabolic disorders, were currently taking oral steroid medications, or were planned to undergo a sub-lobar resection. Informed consent was obtained in clinic during a pre-operative appointment (IRB: UCMC 20-1696). Patient demographics including age, sex, race/ethnicity, comorbidities, frailty status, and pathologic and radiologic evaluation of tumors were recorded **(Supplemental Figure 1F; Supplemental Table 1)**.

Intraoperative sampling

At time of oncologic resection, after the dissection of the hilar vessels, blood samples were obtained by the thoracic surgery team from the pulmonary vasculature and by the anesthesiology team from the systemic circulation. Using a 25 gauge needle, arterial inflow to the lung (the pulmonary artery) and venous outflow from the cancer containing lobe's pulmonary vein and an adjacent healthy lobe's pulmonary vein were sampled **(Supplemental Figure 1A-B)**. Simultaneously, the anesthesiology team obtained blood samples from a radial arterial line and a peripheral vein to provide an approximation of the concentration gradient change of metabolites across the systemic circulation.

Validation of sample processing

A single healthy volunteer provided plasma samples for evaluation of whether plasma metabolite composition is altered by time spent on ice prior to sample processing. A 20 gauge butterfly needle was used to collect 5 mL of blood from the antecubital vein of the volunteer. Four 1cc aliquots

were placed into heparin coated cryovials. Samples were left on ice for 4, 15, 25 and 36 min prior to being centrifuged at 850 x g for 10 min at 4 °C. Plasma was isolated and partitioned into 50 μL aliquots in uncoated eppendorf tubes prior to flash freezing in liquid nitrogen and stored at -80 °C prior to analysis by liquid chromatography/mass spectrometry (LC/MS).

Initial processing of intraoperative samples

In the operating room, blood samples were partitioned into 1 mL aliquots in EDTA coated cryovials before being centrifuged at 800 x g for 10 min at 4 °C. Plasma was partitioned into aliquots of 100 μL into 1.5 mL uncoated eppendorf tubes and flash frozen with dry ice prior to transport and storage at -80 °C prior to analysis by LC/MS.

LC/MS analysis of polar metabolites

Metabolite quantification in human fluid samples was performed as described previously (1). In brief, 5 μL of sample or external chemical standard pool (ranging from ~5 mM to ~1 μM) was mixed with 45 μL of acetonitrile:methanol:formic acid (75:25:0.1) extraction mix including isotopically labeled internal standards. All solvents used in the extraction mix were HPLC grade. Samples were vortexed for 15 min at 4 °C and insoluble material was sedimented by centrifugation at 16,000 x g for 10 min at 4 °C. 20 μL of the soluble polar metabolite extract was taken for analysis of polar metabolites by LC/MS.

Metabolite profiling by LC/MS was conducted on a QExactive benchtop orbitrap mass spectrometer equipped with an Ion Max source and a HESI II probe, which was coupled to a Dionex UltiMate 3000 HPLC system (Thermo Fisher Scientific). External mass calibration was performed using the standard calibration mixture every 7 days. An additional custom mass calibration was performed weekly alongside standard mass calibrations to calibrate the lower end of the spectrum (m/z 70-1050 positive mode and m/z 60-900 negative mode) using the standard

calibration mixtures spiked with glycine (positive mode) and aspartate (negative mode). 2 μL of each sample was injected onto a SeQuant® ZIC®-pHILIC 150 x 2.1 mm analytical column equipped with a 2.1 x 20 mm guard column (both 5 mm particle size; EMD Millipore). Buffer A was 20 mM ammonium carbonate, 0.1% ammonium hydroxide; Buffer B was acetonitrile. The column oven and autosampler tray were held at 25 °C and 4 °C, respectively. The chromatographic gradient was run at a flow rate of 0.150 mL min-1 as follows: 0-20 min: linear gradient from 80-20% B; 20-20.5 min: linear gradient form 20-80% B; 20.5-28 min: hold at 80% B. The mass spectrometer was operated in full-scan, polarity-switching mode, with the spray voltage set to 3.0 kV, the heated capillary held at 275 °C, and the HESI probe held at 350 °C. The sheath gas flow was set to 40 units, the auxiliary gas flow was set to 15 units, and the sweep gas flow was set to 1 unit. MS data acquisition was performed in a range of m/z = 70–1000, with the resolution set at 70,000, the AGC target at $1x10^6$, and the maximum injection time at 20 msec.

Following LC/MS analysis, metabolite identification was performed with XCalibur 2.2 software (Thermo Fisher Scientific) using a 5 ppm mass accuracy and a 0.5 min retention time window. For metabolite identification, external standard pools were used for assignment of metabolites to peaks at given m/z and retention time, and absolute metabolite concentrations determined as previously described (1). To account for individual patient differences, metabolite concentrations in the pulmonary veins (cancer-bearing lobe draining pulmonary vein: PVC; non-cancer-bearing lobe (healthy)-draining pulmonary vein: PVH) were normalized to the patient-specific pulmonary artery (PA) concentration. This was achieved by calculating the difference between each vein's concentration and the corresponding pulmonary artery concentration before comparing the relative consumption and production of individual metabolites.

LC/MS lipidomics

Positive ion mode analyses of polar and nonpolar lipids were conducted using an LC/MS system composed of a Shimadzu Nexera X2 U-HPLC (Shimadzu) coupled to an Exactive Plus orbitrap mass spectrometer (ThermoFisher Scientific). 10 µL of human fluid sample was precipitated with 190 µL of isopropanol containing 1,2-didodecanoyl-sn-glycero-3-phosphocholine (Avanti Polar Lipids) as an internal standard. After centrifugation, 2 µL of supernatant was injected directly onto a 100 × 2.1 mm, 1.7-µm ACQUITY BEH C8 column (Waters). The column was eluted isocratically with 80% mobile phase A (95:5:0.1 v/v/v 10 mM ammonium acetate/methanol/formic acid) for 1 min followed by a linear gradient to 80% mobile phase B (99.9:0.1 v/v methanol/ formic acid) over 2 min, a linear gradient to 100% mobile phase B over 7 min, then 3 min at 100% mobile phase B. Mass spectrometry analyses were performed using electrospray ionization in the positive ion mode using full scan analysis over 220 to 1,100 m/z at 70,000 resolution and 3 Hz data acquisition rate. Other mass spectrometry settings were as follows: sheath gas 50, in source collision-induced dissociation 5 eV, sweep gas 5, spray voltage 3 kV, capillary temperature 300°C, S-lens RF 60, heater temperature 300°C, microscans 1, automatic gain control target $1x10^6$, and maximum ion time 100 ms. Lipid identities were determined on the basis of comparison to reference standards and reference plasma extracts and were denoted by the total number of carbons in the lipid acyl chain(s) and total number of double bonds in the lipid acyl chain(s). Ion counts normalized to an internal standard were reported.

Inductively coupled plasma mass spectrometry (ICP-MS) for metal analysis

Samples were digested with 2 mL/g total wet weight nitric acid (Trace metal grade; Fisher) for 24 h at room temperature (RT), followed by treatment with 1 mL/g total wet weight hydrogen peroxide (Trace metal grade; Fisher) for another 24 h at RT. Samples were diluted with ultrapure water (VWR Chemicals ARISTAR ULTRA) followed by ICP-MS (Perkin Elmer Nexion 2000) using 50 ppb Bismuth as internal standard.

Statistics

Initial descriptive statistical analysis including comparison of individual metabolite concentrations, and linear regression comparing metabolite concentration to patient characteristics including tumor size and PET avidity was performed in R version 4.2.0 (Foundation for Statistical Computing, Vienna, Austria). Metaboanalyst 6.0 (2) was used to perform pairwise comparisons of metabolite concentrations, partial least squares determinant analysis, create metabolite heat maps, and identify variables of importance.

Study approval

Written informed consent was obtained from all patients. This study was initially approved on 5/10/2021 and was overseen for the duration of the study protocol by the University of Chicago IRB (IRB#20-1696). All Intraoperative images were obtained with patient consent at time of their procedure and consent has been retained.

Data availability

Values for all data points in graphs are reported in the Supporting Data Values file. Any additional information required to reanalyze the data reported in this paper is available from the lead contact upon reasonable request.

Author contributions

Johnathan R. Kent (Co–first author)[#]
Conceptualization; Methodology; Investigation; Data Curation; Formal Analysis; Writing – Original Draft; Writing – Review & Editing; Project Administration

Keene L. Abbott (Co–first author)[#]
Methodology; Investigation; Data Curation; Formal Analysis; Visualization; Writing – Original Draft; Writing – Review & Editing

Kent and Abbott contributed equally to this manuscript, order was decided based on time on the project.

Rachel Nordgren
Formal Analysis; Writing – Review & Editing

Amy Deik
Investigation; Writing – Review & Editing

Nupur K. Das
Investigation; Writing – Review & Editing

Millenia Waite
Investigation; Writing – Review & Editing

Tenzin Kunchok
Investigation; Writing – Review & Editing

Anna Shevzov-Zebrun
Investigation; Writing – Review & Editing

Nathaniel Christiansen
Investigation; Writing – Review & Editing

Amir Sadek
Investigation; Writing – Review & Editing

Darren S. Bryan
Investigation; Writing – Review & Editing

Mark K. Ferguson
Investigation; Writing – Review & Editing

Jessica S. Donington
Investigation; Writing – Review & Editing

Alexander Muir
Investigation; Writing – Review & Editing

Yatrik M. Shah
Investigation; Writing – Review & Editing

Clary B. Clish
Investigation; Writing – Review & Editing

Matthew G. Vander Heiden (Co–senior author)*
Conceptualization; Methodology; Supervision; Formal Analysis; Writing – Original Draft; Writing – Review & Editing

Maria Lucia L. Madariaga (Co–senior author)*
Conceptualization; Methodology; Investigation; Supervision; Formal Analysis; Writing – Original Draft; Writing – Review & Editing

Peggy P. Hsu (Co–senior author)*
Conceptualization; Methodology; Supervision; Formal Analysis; Writing – Original Draft; Writing – Review & Editing

Funding

This work is the result of NIH funding, in whole or in part, and is subject to the NIH Public Access Policy. Through acceptance of this federal funding, the NIH has been given a right to make the work publicly available in PubMed Central.

Acknowledgements

The authors thank the patients who participated in this research. K.L.A. acknowledges support from the National Science Foundation (DGE-1122374) and National Institutes of Health (NIH) (F31CA271787, T32GM007287). Y.M.S. acknowledges support from the NIH (R01CA148828, R01CA245546). M.G.V.H. acknowledges support from the MIT Center for Precision Cancer Medicine, the Ludwig Center at MIT, and the NIH (R35CA242379, P30CA1405141). M.L.L.M. acknowledges support from the Respiratory Health Association. P.P.H acknowledges support from the Rogel Cancer Center at the University of Michigan, the Judith Tam ALK Research Initiative, the Lung Cancer Research Foundation, the Burroughs Wellcome Fund Career Award for Medical Scientists, and the NIH (K08CA286759).

References

1. Sullivan MR, et al. Quantification of microenvironmental metabolites in murine cancers reveals determinants of tumor nutrient availability. *eLife*. 2019;8:e44235.
2. Pang Z, et al. MetaboAnalyst 6.0: towards a unified platform for metabolomics data processing, analysis and interpretation. *Nucleic Acids Res*. 2024;Jul 5;52(W1):W398-W406.
